# Recent trends in carbon nanotube (CNT)-based biosensors for the fast and sensitive detection of human viruses: a critical review

**DOI:** 10.1039/d2na00236a

**Published:** 2022-11-09

**Authors:** Hicham Meskher, Hussain Chaudhery Mustansar, Amrit Kumar Thakur, Ravishankar Sathyamurthy, Iseult Lynch, Punit Singh, Tan Kim Han, Rahman Saidur

**Affiliations:** a Department of Process Engineering, Kasdi-Merbah University Ouargla 30000 Algeria hicham.meskher@g.enp.edu.dz; b Department of Chemistry and Environmental Science, New Jersey Institute of Technology Newark 07102 NJ USA; c Department of Mechanical Engineering, KPR Institute of Engineering and Technology Arasur Coimbatore 641407 Tamil Nadu India amritt1@gmail.com; d School of Geography, Earth and Environmental Sciences, University of Birmingham Edgbaston Birmingham B15 2TT UK I.Lynch@bham.ac.uk; e Institute of Engineering and Technology, Department of Mechanical Engineering, GLA University Mathura Uttar Pradesh 281406 India; f Research Centre for Nano-Materials and Energy Technology (RCNMET), School of Engineering and Technology, Sunway University No. 5, Jalan Universiti, Bandar Sunway Petaling Jaya 47500 Malaysia saidur@sunway.edu.my; g Mechanical Engineering Department, King Fahd University of Petroleum and Minerals Dhahran 31261 Saudi Arabia; h Interdisciplinary Research Center for Renewable Energy and Power Systems (IRC-REPS), King Fahd University of Petroleum and Minerals Dhahran 31261 Saudi Arabia

## Abstract

The current COVID-19 pandemic, with its numerous variants including Omicron which is 50–70% more transmissible than the previously dominant Delta variant, demands a fast, robust, cheap, and easily deployed identification strategy to reduce the chain of transmission, for which biosensors have been shown as a feasible solution at the laboratory scale. The use of nanomaterials has significantly enhanced the performance of biosensors, and the addition of CNTs has increased detection capabilities to an unrivaled level. Among the various CNT-based detection systems, CNT-based field-effect transistors possess ultra-sensitivity and low-noise detection capacity, allowing for immediate analyte determination even in the presence of limited analyte concentrations, which would be typical of early infection stages. Recently, CNT field-effect transistor-type biosensors have been successfully used in the fast diagnosis of COVID-19, which has increased research and commercial interest in exploiting current developments of CNT field-effect transistors. Recent progress in the design and deployment of CNT-based biosensors for viral monitoring are covered in this paper, as are the remaining obstacles and prospects. This work also highlights the enormous potential for synergistic effects of CNTs used in combination with other nanomaterials for viral detection.

## Abstract 2

Sensitive and selective detection of SARS-CoV-2 using carbon nanotube (CNTs)-based biosensors for fast, robust and cheap diagnosis of infection to support pandemic management.

## Graphical abstract

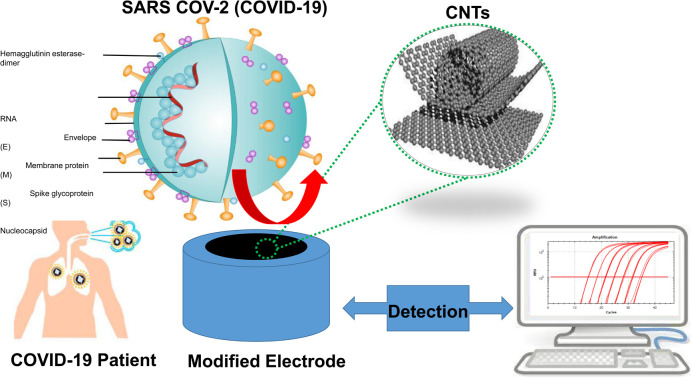

## 1. Introduction

In December 2019, the novel coronavirus (nCoV-2019) pandemic was reported in China; the virus causes severe respiratory disease.^1,2^ It was later named severe acute respiratory syndrome coronavirus 2 (SARS-CoV-2) or corona virus disease-2019 (COVID-19).^3^ The rapid transmission of the virus has created a global health emergency; the World Health Organization (WHO) proclaimed COVID-19 a pandemic on March 13th, 2020, and encouraged scientists worldwide to develop an effective plan to fight the pandemic's fast spread, based on large-scale diagnostic testing.^4^ Diagnostic tools for viral detection were developed before and during the first worldwide wave of the pandemic. However, testing improvements and more rigorous clinical and epidemiological validation are still required for many of these research advances. To battle present and future pandemics, there must be worldwide access to testing, and crucially, infection control and diagnostic testing must be tightly interwoven. The diagnostic tools should guide therapy selection and monitor treatment success. According to the WHO, as of mid-March 2022, more than 464.8 million confirmed cases of Covid-19 were recorded and more than 6 million people died from infection with SARS-CoV-2, although it is generally noted that official figures are much lower than actual cases and deaths. The pandemic has altered every area of existence and life.^5–7^ The impact is significant and will be felt for a long time, as countries shift from eradication approaches to co-existence with the pandemic.

Cough, fever, taste and smell loss, and shortness of breath are among the symptoms of the infection,^8,9^ although symptoms are shifting somewhat as mutations arise. The virus has an incubation period ranging from 2 to 7 days with no evident symptoms.^10,11^ As the transmission rate of COVID-19 is rapid and faster than other respiratory viruses, systematic diagnostic techniques must be developed.^12,13^ Indeed, the appearance of the Omicron variant, which is 50–70% more transmissible than the previously dominant Delta variant, makes this need more pressing.^14,15^ As a standard for developing and validating diagnostic approaches, the WHO has developed a guideline called ASSURED (quality-Affordable, Sensitive, Specific, User-friendly, Rapid and robust, Equipment-free, Deliverable).^16^ Furthermore, COVID-19 is considered a novel pandemic in many aspects, and controlling its spread has proven challenging for all nations.^17,18^ One of the main difficulties was that some of the infected people are asymptomatic but can transmit the virus^19^ from person-to-person contact or *via* small droplets emitted when coughing, sneezing, or speaking.^20–22^ As a result, quick and accurate tests for COVID-19 to identify the need for isolation for the safety of others, must be applied. However, existing diagnostic procedures for COVID-19 are costly and time-intensive. According to Lancet laboratories, the reverse transcriptase polymerase chain reaction (RT-PCR) test price is estimated to be around 32 USD while the rapid antigen test price has been reduced to 16 USD by December 2021.

Promising and inexpensive tools based on nanotechnology are emerging, offering new and innovative sensing applications. When compared to bulk materials, nanomaterials have outstanding characteristics such as high conductivity, many active sites, and high adsorption capabilities. As a result, they are used in several applications, including analytical chemistry^24^ using electrochemical sensors and biosensors,^23^ proteomics,^25^ bio-detection/detection,^26^ biotechnology,^27^ nanomedicine,^28^ drug delivery,^29^ gene transfer,^30^ wound healing,^31^ energy,^32^ and environment.^33^ Nanomaterials have enhanced the binding performance^24–27^ and have high potential for implementation into small devices, such as portable electronic devices.^34,35^ As a result, they have tremendous potential to enhance people's lives by early detection of SARS-CoV-2 before the onset of illness, thus reducing the fast transmission of the pandemic.

**Fig. 1:**
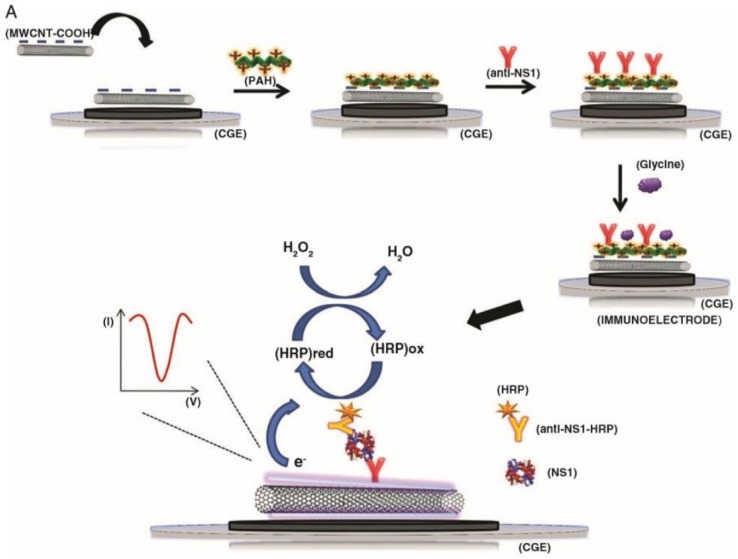
The assembly of a sandwich-type carbon nanotube (CNT) immunosensor and its detection method is depicted schematically. The antibodies are attached onto CNTs through a poly(allylamine) layer. This figure has been adapted/reproduced from ref. 36 with permission from MDPI, copyright 2021.

**Fig. 2:**
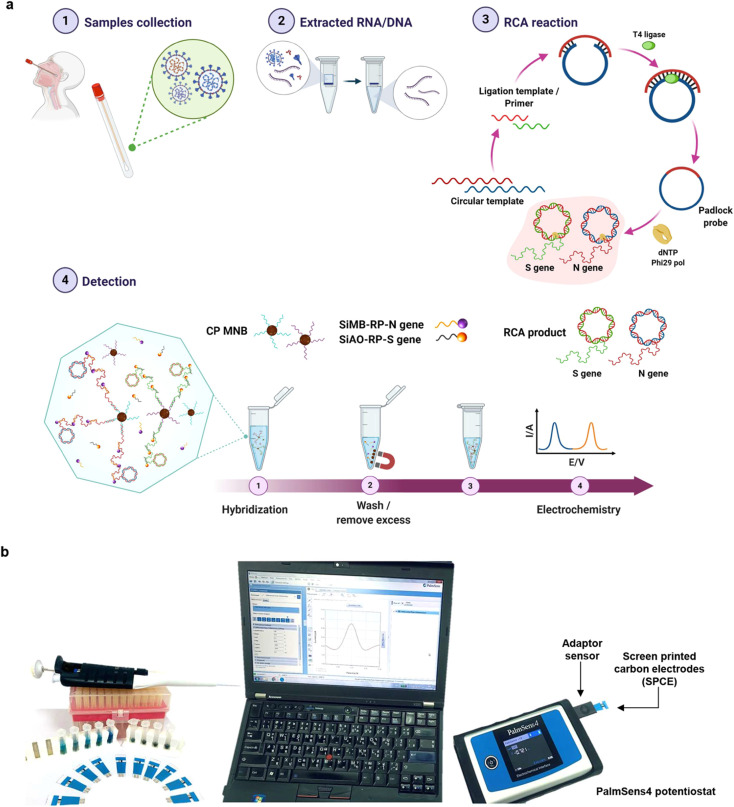
(a) Detection workflow for SARS-CoV-2 using an electrochemical biosensor based on multiplex isothermal rolling circle amplification (RCA) for the fast identification of SARS-CoV-2 N and S genes in clinical samples. Sandwich hybridization of RCA amplicons with probes functionalized with redox-active labels is used in the test, which is then detected using differential pulse voltammetry (DPV). (b) The electrochemical detection setup applied for the study. This figure has been adapted/reproduced from ref. 37 with permission from Nature, copyright 2021.

This article discusses recent breakthroughs in the development of CNT nanocomposites and their combination with other nanomaterials to create selective biosensors to diagnose viral diseases (shown schematically in Fig. 1 and 2), with a focus on COVID-19 diagnosis. After addressing the need for early identification of the infection, the unique characteristics of CNTs to address early detection are presented. Next, the techniques employed for CNT synthesis and some notable breakthroughs in their analytical applications based on several electrochemical techniques, are highlighted. Finally, we provide an overview of the existing challenges and future opportunities for electrochemical biosensors based on CNT nanocomposites to diagnose viral diseases, with an emphasis on SARS-CoV-2 detection.

## 2. Case study: SARS-CoV-2 detection

**Fig. 3:**
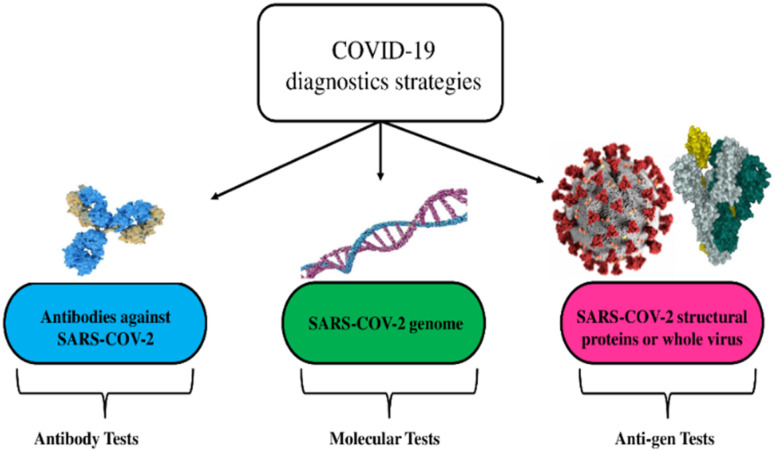
Diagnostic strategies for the detection of viruses are exemplified *via* the coronavirus disease. This figure has been adapted/reproduced from ref. 26 with permission from MDPI, copyright 2021.

Early detection of SARS-CoV-2 is crucial for averting large outbreaks, and many detection approaches (with different modes of detection) are used to balance the need for fast diagnostic strategies with the risk of false positives or negatives.^38–41^ As highlighted in Fig. 3, two primary methods are used to detect the virus: (i) molecular tests based on viral ribonucleic nucleic acid (RNA) or deoxyribonucleic acid (DNA) identification and (ii) serological tests based on the virus or its protein identification (antigen tests), or its antibodies (antibody tests).^42–46^

Molecular tests are costly, time-consuming (results are ready in 2–3 hours), and need lab processing.^47–49^ RT-PCR diagnostics, on the other hand, has a high sensitivity for detecting the virus, with an estimated limit of detection (LOD) of 1 to 10 copies of the viral RNA.^50,51^ Serological assays detect immunoglobulin G (IgG) and immunoglobulin M (IgM) antibodies and give basic monitoring information regarding previous viral infections. As a result, the ideal approach for detecting active SARS-CoV-2 infection is the molecular test for viral RNA detection. However, the procedure's intricacy requires the employment of costly tools and expert staff, limiting testing capacity in developing countries.^52^ Antibody identification is a serological technique that, unlike molecular technologies, can confirm a patient's previous infection. These tests rely on detecting the host's response, which is antibody production against SARS-CoV-2 S proteins. Because viral RNA detection takes a long time to process and has equipment constraints, substantial efforts have been undertaken to propose alternative targets and methodologies for viral testing to deliver simpler and more efficient diagnostics. Furthermore, antibody and antigen testing need specialized, expensive optical imaging equipment as well as requiring a long time to process, as mentioned earlier, leading to a long run time.

There are certain limitations to traditional molecular and serological approaches. Sample preparation and cold storage are essential for such tests, which necessitate sophisticated and costly equipment. The serological test eliminates some of these limitations, as these tests are sensitive to the late and recovery stages of infection, which is of great value to identify infected individuals, but they face the challenge of low accuracy toward SARS-CoV-2 detection. On the other hand, as advised by UK authorities, lateral flow immunoassay technology has been introduced due to its low cost, low detection limit, and rapid and sensitive detection. In the rapid propagation of the pandemic, lateral flow technology might play a crucial role in the fastest identification and effective isolation of infected people.^53^ Thousands of lateral flow test kits have been created. However, the need for careful examination of sensitivity and specificity restricts its usage, because in regions where testing and isolation is a point of first defense, false-negative findings would worsen the situation by spreading the virus further,^54^ as happened in Liverpool in December 2020, when lateral flow testing missed more than half of the infection instances.^55^

The diagnosis of COVID-19 infection is frequently mistaken with other viral infections. To mitigate misunderstanding and incorrect outcomes, accurate and rapid diagnosis is required. To this purpose, the next section of this study sheds light on the constantly expanding body of present and in-development diagnostic assays, especially electrochemical biosensors, which are based on nanomaterials, CNTs in particular.

## 3. Nanomaterials and the need for biosensors to detect viruses

Due to the long operating time and equipment complexity involved with viral RNA detection and identification, there has been a significant need to identify alternative viral test targets and procedures for simpler and faster diagnostics.^56^ Nanomaterial-based analytical tools offer viable alternatives to RT-PCR for rapid and accurate virus detection.

**Fig. 4:**
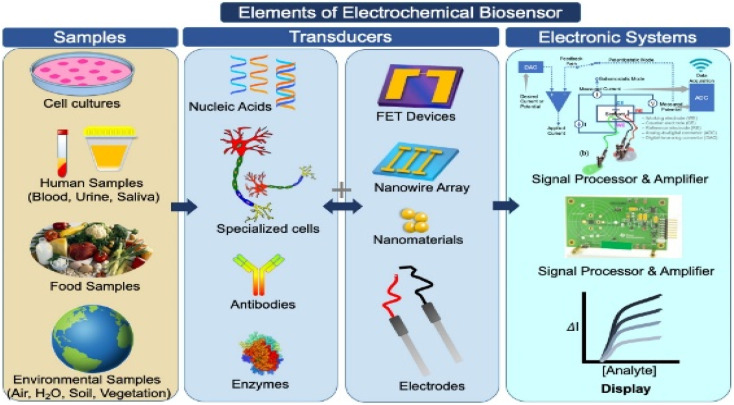
Illustration of the design elements of biosensors used to detect target test samples, with an emphasis on the electrochemical bio-sensing platforms, which translate biochemical data into current or voltage signals on the surface of an electrochemical biosensor. This figure has been adapted/reproduced from ref. 23 with permission from Elsevier, copyright 2021.

As a result, scientists are seeking a low-cost, fast, and simple method to detect SARS-CoV-2 efficiently, with biosensors emerging as the best instruments for detecting SARS-CoV-2 efficiently and precisely. Biomolecule detection is carried out with the help of a biosensor, which is made up of two parts: a biomolecule receptor and a physicochemical transmitter.^57,58^ A typical biosensor architecture is described in Fig. 4, which contains biometric recognition components, an electrode-active surface, and a data processor. The physiologically sensitive substance serves as a detection pattern for the sensor. The bioanalyzer's contact with the second receptor is transformed into an electrical signal which will be appropriately amplified before being output.

The biomolecule-receptor is responsible for determining the quantities of the studied analytes, such as nucleic acids (DNA and RNA), antibodies, and cell receptors, bound to the biosensor. A biochemical signal is produced by a bio-molecular analysis process which will be amplified into quantifiable data before being processed for input into the display device.

Seo *et al.*, for example, created a field-effect transistor sensor by activating graphene with an anti-COVID-19 protein antibody to monitor COVID-19 at a level of 242 copies per ml of biofluid.^59^ Several potential SARS-CoV-2 nanotechnology-based biosensors for DNA- and antigen-based COVID-19 diagnosis have also been developed, utilizing carbon-based nanomaterials, quantum dots, metals, and metal oxide nanomaterials.^60–63^ For example, a study published by Wang *et al.*^60^ demonstrated the sensitive detection of SARS-CoV-2 using a lateral flow immunoassay based on ‘Dual-Mode’ colorimetric and fluorimetric sensing using quantum dot nanobeads (NBs) (SiO_2_@Au@QD NBs); these innovative nanomaterials can be used in the colorimetric mode for rapid instrument-free screening of infected patients, *versus* in the fluorescent mode for the sensitive and quantitative determination of specific IgM/IgG concentrations in blood samples. A biosensing platform that enables multiple re-uses of the device *via* rapid regeneration at low pH has also been reported, which was created by using 3D printed electrodes, obtained *via* coating the electrodes with reduced graphene oxide (rGO) nanoflakes and immobilizing specific viral antigens onto the rGO nanoflakes.^64^

**Fig. 5:**
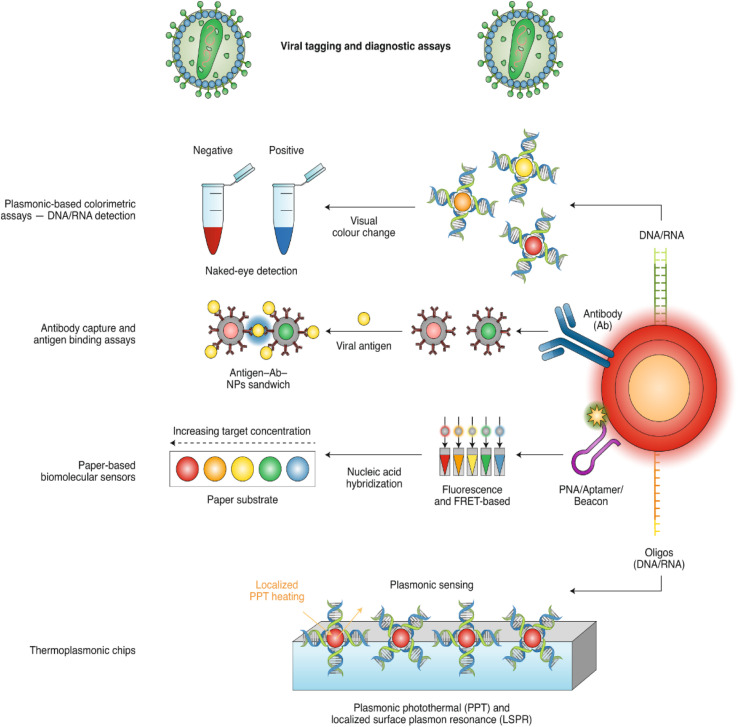
Nanomaterials functionalized with nucleic acids or antibodies represent the main strategies of nano-based detection. This figure has been adapted/reproduced from ref. 82 with permission from Nature, copyright 2020.

**Fig. 6:**
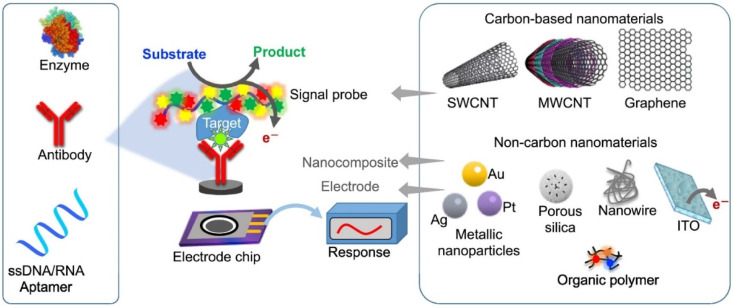
Schematic illustration of the analytical principle of electrochemical biosensors. This figure has been adapted/reproduced from ref. 83 with permission from Springer, copyright 2020.

Biomolecule sensors will be required to create sensitive assays that do not rely on specialist equipment to interpret signals or controlled laboratory settings to handle samples.^65,66^ The capacity to perform rapid and ultra-sensitive measurements with tiny quantities of analyte is one of the advantages of biosensors.^67,68^ Since the creation of the first biosensor,^69^ a diverse range of biosensors have been the subject of research and development covering a wide range of applications, such as glucose biosensors, for example. However, despite significant advances and investment, viral biosensors are not yet commercially available. In parallel, due to their novel and unique features, nanomaterials have piqued the interest of many researchers since their discovery.^70–74^ Therefore, various types of nanomaterials, such as gold and other metal-based nanomaterials, have been used to prepare lab-scale biosensors for rapid, easy-to-use, robust, and low-cost detection of viruses. As shown in Fig. 5 and 6 various viruses, including HIV, acquired immunodeficiency syndrome (AIDS),^75^ hepatitis B virus,^76^ influenza virus,^77^ and herpes virus,^78^ have been effectively detected using nanomaterial-based biosensors. For the sensitive detection of the SARS-CoV-2 virus, detection systems based on gold nanostructures, nanoparticle-activated magnetic detection, and biodetection systems based on lanthanide nanoparticles (NPs)^72^ have recently been described. Various biosensors based on nanomaterials have been reported to detect the SARS-CoV-2 RNA, antigen, or antibody within 10 to 100 minutes.^79–81^

As summarized in Table 1, arc-discharge,^84^ plasma-enhanced chemical vapor deposition (CVD),^85^ laser ablation,^86^ chemical vapor deposition,^87^ and the floating catalyst method^87^ are the most used methods to synthesize CNTs. In this context, the catalytic CVD technique, in particular the floating catalyst method, is considered to be the best way to generate a large number of CNTs efficiently with high purity. Compared to arc discharge and other techniques, this technology is more controlled and cost-effective while preparing small amounts of CNTs. In addition, simple, low energy consumption and cost-effective synthesis procedures have been recently developed to reduce the cost of CNT production.^88–90^ The expense of developing bio-sensing platforms is a big worry in a pandemic scenario like COVID-19, as millions of tests must be conducted every day. As highlighted in Table 2, CNTs are a great contender for making high-performance and low-cost detection systems in the laboratory due to their low price. However, when preparing large amounts of such sensors, their price should be taken into account and must be further optimized. As they are mass produced, CNTs are inexpensive compared to many other nanomaterials, however, relative to other components of biosensors they can be a major contributor to the overall cost of the sensors.

**Table 1:** Summary and comparison of the most important synthesis procedures for CNTs

Synthesis method	Principle	SWCNTs or MWCNTs	Reference
Arc-discharge	An electric arc discharge between two electrodes at *T* > 3000 °C causes carbon to sublimate from graphite. In the presence of appropriate metal catalyst particles (Fe, Co, or Ni), nanotubes develop	Both	91
Laser-ablation	The laser-ablation technique creates CNTs by irradiating a graphite rod with catalysts heated to 1000 °C or more with a pulsed laser	SWCNT	92
Chemical vapor deposition (CVD)	At high temperatures (500–1000 °C), metal nanoparticles (Co and Fe) accelerate the decomposition of a gaseous hydrocarbon source (ethylene or acetylene)	Both	93 and 94
	At high temperatures, carbon has poor solubility in certain metals, causing precipitation & formation of nanotubes		
Plasma-enhanced chemical vapor deposition (PECVD)	PECVD involves the creation of a glow discharge in a chamber. Thermal CVD requires temperatures from 500–1000 °C. This technique is highly adaptable, but has two drawbacks: first, the tubes are not correctly oriented or in the ideal straight form. Second, the substrate material is destroyed by high temperatures	Both	95 and 96
Floating catalyst (FC)	Floating catalytic chemical vapor deposition (FC-CVD), also known as aerosol-aided FC-CVD, uses an ultrasonic bath to keep the solution homogeneous outside the reactor, which controls the size, quality, and the purity of the CNTs produced	Both	97 and 98

**Table 2:** Comparison of the price of CVD and electric arc discharge synthesis of CNTs

Carbon nanomaterials	Diameter or size (nm)	Method	Assay	Price	Source
SWCNTs	0.7–1.1	CVD	>77%	$1600 per g	https://www.sigmaaldrich.com (July 2021)
	0.7–0.9		>93%	$1500 per g	
	—		>95%	$1650 per g	
	4.0–5.0	Electric arc discharge	>90%	$2720 per g	
	4.0–5.0		>80%	$690 per mg	
MWCNTs	110–170	CVD	>90%	$80 per g	
	6–13		>98%	$100 per g	
	7–15	Electric arc discharge	>20%	$920 per g	

CNTs have exceptional physical and chemical properties, such as high electrical conductivity, large surface area to volume ratio, chemical consistency, and mechanical strength. As a result, CNT-based detection systems have grown quite popular in recent years,^99,100^ with researchers globally utilizing them to identify pathogens^101^ and viral pathogens,^102,103^ and vitamins with ultra-high sensitivity and specificity.^104^

## 4. CNT-based electrochemical biosensors for medical applications

When a biological recognition component is immobilized on an electrochemical cell's electrode it is called a biosensor. An electrical signal will be recorded when the biological sample interacts with the biological recognition site of the biosensor. If at all feasible, the biological sensing matrix should be placed on top of a switching conductive material with strong electron transfer capacity, and a large surface area to capture the analyte of interest. Since CNTs possess all these characteristics, CNT-based biosensors^105,106^ have been used successfully in laboratory testing for respiratory virus detection.

Ma *et al.*^107^ developed a simple and sensitive method to identify human immunodeficiency virus p24 (HIV-p24) using a glassy carbon electrode (GCE) modified by surface polymerization. The proposed biosensor-based-molecularly imprinted polymers showed specific recognition of HIV-p24 and displayed a LOD of 0.083 pg cm^−3^ (S/N = 3). An immunosensor for antibody detection directed against the hepatitis B central protein (anti-HBc) was developed based on a hybrid hyaluronic acid-CNT film.^108^ The immunosensor response was linear towards anti-HBc at concentrations up to 6 ng ml^−1^, with a LOD of 0.03 ng ml^−1^. In a similar study, a novel immune electrochemical sensor based on a 3D array of carbon nanotube-conductive polymer (CNT-CP) was reported to detect hepatitis B surface antigen (HBsAg) in human serum. The CNT-CP nanocomposite was created by drop-casting CNT solution onto a GCE, followed by electrochemical polymerization of poly(propionic pyrrole) for crosslinking and stabilization of the CNTs.^109^ Carbon nanotubes are often used in electrochemical biosensors because their high surface-to-volume ratio leads to large numbers of defects (where the carbon atoms have sp^3^ hybridization or there is an absence of some sp^2^ carbon atoms) that serve as electrically active sites for heterogeneous electron transport.^110–112^

A favorable combination of molybdenum disulfide (MoS_2_) material and CNT aerogel has been produced by Tain and colleagues.^113^ The active sites produced by the three-dimensional CNTs/MoS*x* aerogel were sufficient for fast charge transmission. The suggested immunosensor detected the avian influenza virus H7 (AIV H7) sensitively. The immunosensor, which used PAb-CNTs/MoS*x* as the identification antibody, had a wide linear range of 1–25 ng ml^−1^, while the detection limit was estimated to be 0.43 ng ml^−1^. Bonanni *et al.*, proved that CNT-based biosensors may be applicable to detect human influenza (H1N1)^114^ with a low LOD of 7.5 fM (corresponding to 577 pM). In addition, electrochemical detection without labeling of the avian influenza virus genotype using an MWCNT-cobalt phthalocyanine-polyamidoamine (PAMAM) nanocomposite-modified GCE has been proposed by Zhu *et al.*^115^ In a similar study, Fu and co-authors developed a chemoresistance-like biosensor based on CNTs for highly efficient and rapid detection of DNA sequences of the avian influenza virus (AIV) subtype H5N1. The manufactured biosensor could reliably detect target DNA sequences complementary to AIV H5N1.^116^ For the detection of the dengue virus, Silva *et al.*^117^ used a poly(allylamine)/CNT electrochemical biosensor to detect the virus down to the level of 0.035 μg ml^−1^. Molecularly imprinted polymers (MIPs) combined with an MWCNT-based-biosensor for the sensitive identification of HIV-p24 and other infectious diseases were proposed by Ma *et al.*^107^ and Cui *et al.*^118^ In a study by Lu and co-workers, a flexible paper-based electrode to detect HIV was developed; the sensor is based on a nickel-metal organic framework (Ni-MOF) with AuNPs deposited onto carbon nanotubes/polyvinyl alcohol (Ni-MOF-Au/CNT/PVA) in a cellulose membrane, for the detection of human immunodeficiency virus (HIV) DNA^119^. The Ni-MOF-Au/CNT/PVA–based electrode allows higher loading of the probe DNA than the CNT/PVA electrode, which improves the sensitivity of the electrode. Before and after hybridization with HIV DNA, the variability of the methylene blue peak current adsorbed onto the DNA was measured. The electrochemical results showed that even after 200 repetitions of bending or stretching under various loads, ranging from 0% to 20%, the Ni-MOF-Au/CNT/PVA film retained its electrochemical properties. With a linear range of 10 nM to 1 M and a LOD of 0.13 nM, the flexible paper electrode demonstrated high performance and good HIV-DNA detection.^119^

In a related study, 3D CNT/MoS*x* aerogels were used to create a sandwich-type electrochemical sensor for the sensitive detection of the influenza virus, utilizing Avian H7 (AIV H7).^120^ CNT/MoS*x* aerogels were made using a simple thermal technique that involved dissolving a combination of (NH_4_)_2_MoS_4_(NH_4_) and CNT aerogels in dimethylformamide (DMF) and heating it at 200 °C for 10 hours, ensuring that the CNTs and aerogel structures fit together firmly, providing an overlapping layered structure that is critical for securely connecting the MoS*x* membranes to the CNT antenna electrode surface. To make the PAb-AuNPs-CNTs/MoS*x* bio-conjugates, 10 ml of 1-Ethyl-3-(3-dimethylaminopropyl)carbodiimide (EDC) solution and 20 ml of nitrogen-hydroxysuccinimide (NHS) solution were combined in phosphate buffer solution (PBS) (pH 5.2) and used to activate 400 ml of AuNPs-CNTs/MoS*x* (0.1 mg ml^−1^) for 30 minutes. The nanohybrid generated enough active sites for rapid charge transmission, and detection of AIV H7 was achieved through adsorption of the polyclonal antibody H7 (PAb-CNT/MoS_2_) onto the modified surface of the CNTs/MoS*x*. With PAb-CNTs/MoS*x* as the detection antibody, the immunosensor had a linear range from 1 to 25 ng ml^−1^ and an LOD of 0.43 ng ml^−1^. The large surface area of the 3D carbon materials demonstrated higher electrical conductivity compared to ordinary carbon materials, which is favorable for electron transport.^120^

Fu *et al.* also developed a CNT-based biosensor for the effective and rapid detection of the H5N1 subtype of the avian influenza virus.^121^ As alternate active sensing components, semiconducting single-walled carbon nanotubes (sc-SWCNTs) or nitrogen-saturated multi-walled carbon nanotubes (N-MWCNTs) were utilized, and their sensitivity to varying concentrations of the DNA target was examined. DNA probe sequences that are not covalently linked to the sidewalls of the nanotubes have been employed. In 15 minutes at ambient temperature, the nanotube sensors can accurately detect the complementary DNA target sequence of VIA H5N1 at concentrations ranging from 2 pM to 2 nM.^121^

Thin SWCNTs were utilized to build a layer-by-layer surface self-assembly porcine biosensor for the influenza virus (H1N) because of their exceptional electrical properties.^122^ The surface absorption of large molecules, such as poly-l-lysine, anti-simian immunodeficiency virus (anti-SIV) antibodies, and SIV tended to enhance immunostaining. Resistance variation (including natural background resistance) during viral binding was balanced by the impedance of the bare devices, extending the detection range while reducing the required channel length of the CNT resistors. This biosensor was proposed to be potentially useful as a point-of-care detection tool or as the basis of a lab-on-a-chip system since its limit of detection toward the influenza virus (SIV) was estimated to be 180 TCID_50_ per ml (TCID_50_: 50% tissue culture infectious dose). Portable antibody-based assays can also be employed in conjunction with microfluidic/nanofluidic systems for the clinical diagnosis of SIV H1N1.^122^

The antiviral medicine Daclatasvir (DAC) is on the WHO list of essential pharmaceuticals for a basic health system, and as such, electrochemical and impedance spectroscopy approaches are used to learn more about its mechanism of action. For example, a carbon paste electrode (CPE) modified with chitosan was developed for biosensing through immobilization of positively charged biomolecules, such as the hepatitis B virus.^123^ Differential pulse voltammetry (DPV) was used to explore the simultaneous detection of DAC at pH 2.0 in a bulk buffer. The results show a linear connection between the DAC current peak and its concentration in the range of 1.0 nM to 12 mM while the LOD was estimated to be around 0.882 nM. The concentration of DAC in pharmaceutical formulations, and human biological fluids including urine and blood serum, and in the presence of co-administered medicines could be measured/monitored successfully, with the sensor displaying an intriguing qualitative capability as well as a long lifetime, opening up new possibilities for future applications.^123^

CNT-based sensors have been demonstrated to effectively detect biomolecules such as proteins, RNA, immune-active chemicals, and lectins. At the laboratory scale, CNT-based sensors have demonstrated improved repeatability, sensitivity, dependability, and cost-effectiveness compared to other nanomaterials such as graphene and metal oxide nanoparticles.^26,83^

## 5. SWCNT- and MWCNT-based biosensors for SARS-CoV-2 detection

Various research groups developing SWCNT- and MWCNT-based biosensors have reported success in the diagnosis of COVID-19.^124–126^ SWCNTs and MWCNTs can be utilized to obtain improved biocompatibility with biological components such as RNA and DNA while creating biosensors with acceptable repeatability, as previous studies have reported.^127^ There have been notable advances in the use of biosensors for the detection of SARS-CoV-2; as biosensors provide good selectivity, as well as producing ultra-sensitive measurements with limited concentrations of analytes, they are becoming increasingly popular.^128,129^

Thanihaichelvan *et al.*^124^ proposed and demonstrated a novel biosensor for the selective detection of SARS-CoV-2 based on a CNT-FET. The sensor was created by inactivating the reverse sequence of the RNA-dependent SARS-CoV-2 polymerase gene on the channel and manufacturing CNT FETs on a flexible Kapton substrate. The biosensor showed a positive target sequence selective detection response with a detection limit of 10 fM. The promising findings suggest that CNT FET-based biosensors could be used as a diagnostic tool for COVID-19. Likewise, Shao *et al.*^130^ described how they used a SWCNT-based semiconductor FET to detect SARS-CoV-2 antigens. SWCNT FET sensors were designed by activating the anti-SARS-CoV-2 antibody (SAb) protein and anti-cardiac protein antibody, and detecting the S antigen (SAg) and N antigen (NAg), with lower detection limits of 0.55 fg ml^−1^ for SAg and 0.016 fg ml^−1^ for NAg in titration samples compared to PCR tests.^131,132^ When compared to serological testing and test reaction sequencing polymerase, SAb functional FET sensors demonstrated good detection performance in distinguishing between positive and negative clinical samples.^131,132^

Angiotensin-converting enzyme 2 (ACE2), a host protein with strong binding to the COVID-19 S protein, was used by Pinals and co-workers to build a nanosensor based on non-covalently functionalised SWCNTs.^56^ Within 90 minutes of being exposed to the COVID-19 S protein, the fluorescence of the nanosensor increases two-fold. The authors discussed the nanosensor's stability and detection process, as well as the inert nanosensor used to sustain the detection response in saliva as the viral transmission medium. The research also demonstrated that these ACE2-SWCNT nanosensors preserved their detection capabilities in a stable state on the surface, with a fluorescence activation response of 73% within 5 seconds of being exposed to 35 mg l^−1^ of the SARS-CoV-2 virus.

With the outstanding purity of CNTs, which gives high conductance and a high on/off ratio for biosensor preparation, the studies described herein illustrate the most important CNT-based biosensors for analyzing SARS-CoV-2. However, the next emerging research direction will focus more on the combination of the wide range of nanomaterials that have been reported as having good antiviral action, including metals and metal oxide nanoparticles, with CNTs into a single bio-recognition matrix to diagnose COVID-19. The combination of CNTs with other nanomaterials has been applied in different domains such as electrochemical sensors,^133^ biosensors,^134^ biomedical devices^135^ and environmental monitoring.^136^ For example, the synergistic effects of titanium dioxide (TiO_2_) NPs and CNTs are well explored, with this carbon-metal oxide nanocomposite improving the electrochemical transduction due to enhanced electron transfer rates between the analyte and carbon-metal oxide nanocomposite. Hence, the good electrochemical performance of such a combination can be attributed to the excellent electronic conductivity of the functionalized CNTs and the affinity reaction on the engineered-interface-based carboxyl groups in both CNTs and TiO_2_ NPs which are likely to have high interaction with the studied analyte.^133^ Miripour *et al.*^126^ created a real-time electrochemical sensor to detect SARS-CoV-2. In this sensing device, an MWCNT-modified electrode is employed as a selective electrochemical tracer of the reactive oxygen species (ROS) superoxide. The intensity of ROS emission into the sputum may be measured using cyclic voltammetry to capture the produced electrochemical signaling changes due to binding of the ROS. When compared to clinical diagnostic results from more than 140 routine and difficult cases, the prepared sensor exhibited 97% accuracy and sensitivity.^126^

Similarly, Zamzami and coworkers created a rapid and cost-effective electrochemical biosensor based on a CNT-field-effect transistor (CNT-FET) that allows digital detection of SARS-CoV-2 S1 antigens.^137^ The biosensor was created on an Si/SiO_2_ surface using CNT printing and immobilization of anti-SARS-CoV-2 S1. The SARS-CoV-2 S1 antigen was detected by the CNT-FET biosensor at concentrations ranging from 0.1 fg ml^−1^ to 5.0 pg ml^−1^ in 10 mM ammonium acetate (AA) buffer pH 6.0. The developed biosensor demonstrated good selectivity for SARS-CoV-2 S1 antigen detection,^137^ but testing in the sputum or other biofluid was not reported.

Furthermore, using Lennard-Jones potentials, Jomhori *et al.* investigated the affinity of SWCNTs for the B-domain of the S1 subunit of the S-protein. The interaction of SWCNTs with the B-domain was assessed using the 2-acetamido-2-deoxy-β-*d*-glucopyranose group, which revealed a change in the affinity of the S1 subunit, which may prove to be a barrier for viral propagation.^138^ Furthermore, Jeong *et al.*^139^ confirmed that the use of CNTs in viral nucleic acid extraction enables high-yield and high-sensitivity identification of SARS-CoV-2 nucleic acids while depending less on reagents that are vulnerable to change due to supply chain restrictions than other biosensing platforms.^139^

When coupled with CNTs, many other nanomaterials, currently in the early phases of research, may offer fascinating properties in terms of viral detection.^140^ This includes nanosensors for the rapid detection of the SARS-CoV-2,^57^ and nanomaterials that can help in the development of new approaches to immunization.^141^Table 3 highlights some of the recent biosensor advances that have demonstrated promise in detecting SARS-CoV-2.

**Table 3:** CNT-based electrochemical biosensors for the detection of SARS-CoV-2

CNT-based electrochemical biosensors		
Recognition element	LOD	Reference
S protein	—	56
RNA	10 fM	124
ROS	—	126
Spike (S)-antigen (SAg)	0.55 fg ml^−1^	130
Nucleocapsid (N)-antigen (NAg)	0.016 fg ml^−1^	130
S protein	—	138
Viral RNA	—	139
S protein	4.12 fg ml^−1^	137

## 6. Challenges & drawbacks of using CNTs for viral detection

Although biosensors have become an appealing choice for detecting many types of viruses, they do have significant limitations. It is challenging and expensive to synthesize ultrapure carbon nanotubes with similar SWCNT or MWCNT characteristics. Synthesis of CNTs is a sophisticated process and requires skilled operators; any malfunction in the preparation of CNTs will directly affect their characteristics and parameters, then the performance and response characteristics of the biosensor may change.

CNT-based biosensors provide excellent sensitivity and selectivity compared with traditional tests (*e.g.*, PCR and lateral flow tests).^124,142,143^ Hence, these kinds of biosensors can help to confront the challenge of SARS-CoV-2 false-positive and/or false-negative results which may make the spread of the pandemic even worse.^144,145^ CNTs have a wide range of functionalities that can be combined to enable specific human virus detection applications. They can function as selective biosensors sensitive to viral biomarkers, but they are expensive and difficult to manufacture. For example, making biosensors often necessitates a specific size and helical structure. Controlling the size of CNTs during manufacture is very challenging due to the absence of structural control at the atomic level in their synthesis. Current synthetic approaches provide structures with varying physical characteristics.^146^

Although CNT-based biosensors are promising, they still have a wide range of practical challenges to overcome before their widespread application is feasible. For example, it is extremely difficult to attain both low cost and high purity in the mass manufacture of CNTs, which is why current market prices for CNTs are too exorbitant for any practical commercial biosensing application, particularly at the scale needed during the present pandemic. Thus, while CNTs are mass produced and are inexpensive compared to many other nanomaterials, they can be a major contributor to the overall cost of the sensors as they are expensive relative to the costs of other components of biosensors.

For successful CNT-based biosensors, the enzyme must be stationary on the surface of the tubes. However, enzyme immobilization must be performed carefully, since it may impair the enzyme's biological function, biocompatibility, and structural stability. Thus the possible influence of the immobilization strategy on the selectivity and sensitivity of CNT-based biosensors should be taken into account. For sensors that require the integration of CNTs in biological cells and tissues, it is vital to investigate the cytotoxicity of CNTs toward biological species. As a result, there is a pressing need to standardize the structural and surface properties of CNTs, including the defects that have been shown to optimize sensing capabilities, as well as harmonising and streamlining procedures for assessing cytotoxicity. The structural stability and reproducibility of the surface characteristics of CNTs must be increased. Continuous CNT fibers eliminate the need for CNT filtering, and the electrodes may be implanted for *in vivo* testing.^147^ At the moment, CNT-based sensor fabrication and applications are still in the experimental stage; however, if CNT-based biosensors are to be upgraded to commercial clinical use, the fabrication, functionalization, and safety issues must be addressed.

CNTs offer excellent thermal, mechanical, and electrical characteristics, and are mass-producible. However, as shown in Table 2, the cost of producing CNTs is still too high for preparing large numbers of high purity CNT-based sensors currently, but this may change as demand increases. The optimized hybrid-sensing devices require the use of metallic nanoparticles, which might be harmful and hence restricts their possible application. It is crucial to highlight, however, that potential hazards associated with CNT-based biosensors mostly arise from the presence of metal impurities, due to catalysis processes, or from the enhancement of functionality through functionalization with metal/metal oxide nanomaterials, as in many of the examples presented above. As such we strongly recommend that future research focuses on the development and synthesis of CNTs through ecofriendly and green synthesis procedures, and evaluates the potential toxicity of the device components at all stages of their life cycle, including end-of-life considering also the potential for recovery of critical elements, for example.^143,148,149^ Indeed, greener synthesis of nanomaterials has been evaluated using a broad variety of biomolecules, including DNA, proteins, and plant extracts, and for CNTs, various of natural catalysts have been used to functionalize pure CNTs based on eco-friendly synthesis procedures.^150,151^

Sensors need to be both robust and repeatable. Another future challenge will thus be to create durable and repeatable sensors that can be used (and re-used) on real-world samples. For clinical samples, this entails batch manufacturing sensors that produce consistent findings. The sensor may have to be destroyed (*e.g.*, screen-printed electrodes that are cheap enough to be used just once) or reused entirely, and most contemporary designs employ inflexible electrodes, such as GCEs, which do not comply with current standards in place for *in vitro* medical devices (*e.g.*, the European Medicines Agency (EMA) regulations on *In Vitro* Diagnostic Devices (Regulation (EU) 2017/746)).^152^ Alternatively, future research may concentrate on implanted CNTs (although this brings another whole set of safety and efficacy requirements for approval from medical authorities, for example *via* the EMA Medical Devices regulations (Regulation (EU) 2017/745)).^153,154^

Significant work must be done before CNT technology is mass produced for viral detection. Solving the challenges inherent in the development of CNT-based biosensors necessitates collaboration between materials scientists and engineers who build innovative devices such as biosensors, and biologists/virologists.

## 7. Outlook, scope, and conclusions

The world has rarely witnessed as serious a viral pandemic as COVID-19 with its rapid growth and transmission all over the world, putting global health at risk. Thus, the global pandemic has been a significant impetus to accelerate the pace of translation of sensor technologies from lab to clinical use for quick and efficient detection of the corona virus and to keep up with the various mutations that have emerged over the last 18 months. Nanomaterials have enormous promise to address major challenges such as the need for sensitive and rapid detection of emerging viruses due to their unique characteristics that cannot be achieved with bulk materials. Thus, materials science has indeed come to the fore, with special emphasis on nanostructured materials to prepare rapid and sensitive biosensors for SARS-CoV-2 detection.

Despite the emergence of a huge number of novel techniques to address the issues related to the COVID-19 pandemic utilizing various classes of nanomaterials in the scientific literature, this subject is still in its infancy. This review highlighted the most significant studies and mentioned the challenges that need to be overcome to translate the scientific advances into clinically useful devices. The present review summarized the current understanding of the role of CNT-based biosensors in the monitoring of viruses and infectious diseases, including SARS-CoV-2, and attempts to identify other nanomaterials that can be combined with CNTs for this purpose to enhance the diagnostic capability, including for mutations and variants. It also notes the potential of using functionalized CNTs for specific biomolecule immobilization to solve the problem of selectivity and to overcome the challenge of wrong results (false-positive and false-negative results). We actively urge researchers to focus viral diagnostics on CNT-based sensors, which have previously demonstrated high sensitivity and strong selectivity toward respiratory viruses such as SARS-CoV-2.

Based on the results given in the present review, we strongly emphasize the following points to readers:

• The outstanding characteristics of CNTs including mechanical strength, a large surface area to volume ratio, excellent electrical conductance, electrochemical stability in aqueous and non-aqueous samples, and high thermal conductivity, make CNTs an ideal material for ultrasensitive biosensors.

• The development of appropriate methods for tackling the challenges connected with the rapid and sensitive detection of SARS-CoV-2 is now underway in scientific research. However, the path “from laboratory to reality” is difficult and must be improved by increasing the production of high-purity yet low-cost CNTs to facilitate scale-up, and through development of composites with other cheap nanomaterials to improve the uptake of CNT-based sensors and biosensors into commercial use. Thus, the cost of preparation, as well as the stability and reproducibility of CNTs, must be addressed, and better control over their properties is highly recommended.

• To promote the performance and speed up CNT-based biosensors' response to identify SARS-CoV-2, researchers must explore the potential for synergistic effects of CNTs with other nanomaterials such as metals and metal oxides, and their biocompatibility with biological components such as DNA and enzymes. The creation of highly effective immobilized biocatalysts is the short-term objective of lab-scale research, although in order to assess an immobilized enzyme's suitability for scale-up applications for viral detection, critical economic and environmental evaluations of immobilized enzyme-catalyzed processes are needed. For commercial-scale biocatalytic processes in such a setting, techno-economic studies and life-cycle assessments must be examined. On the other hand, a thorough understanding of the economic dynamics that surround process development is still necessary to ensure that enzyme catalysis is implemented in commercial-scale processes in a manner that is acceptable and to promote the creation of optimum enzymes.

## Abbreviations

AA: Ammonium acetate
ACE2: Angiotensin-converting enzyme 2
AIDS: Acquired immunodeficiency syndrome
AIV: Avian influenza virus
anti-HBc: Antigen-hepatitis B central protein
ASSURED: quality-Affordable, Sensitive, Specific, User-friendly, Rapid and robust, Equipment-free, Deliverable
Au: Gold
CNT: Carbon nanotubes
CNT-CP: Carbon nanotube-conductive polymer
COVID-19: Corona virus disease-2019
CPE: Carbon paste electrode
CVD: Chemical vapor deposition
DAC: Daclatasvir
DMF: Dimethylformamide
DNA: Deoxyribonucleic acid
DPV: Differential pulse voltammetry
EDC: 1-Ethyl-3-(3-dimethylaminopropyl)carbodiimide
EMA: European Medicines Agency
FC: Floating catalytic
FC-CVD: Floating catalytic chemical vapor deposition
FET: Field-effect transistor
FRET: Forster resonance energy transfer
GCE: Glassy carbon electrode
H1N1: Human influenza
HBsAg: Hepatitis B surface antigen
HIV: Human immunodeficiency virus
IgG: Immunoglobulin G
IgM: Immunoglobulin M
LOD: Limit of Detection
LSPR: Localized surface plasmonic resonance
MIPs: Molecularly imprinted polymers
MOF: Metal organic framework
MoS_2_: Molybdenum disulfide
MoS_4_: Disulfido(dithioxo)molybdenum
MWCNTs: Multi-walled carbon nanotubes
NAg: N antigen
NBs: Quantum dot nanobeads
nCoV-2019: Novel corona virus
NH_4_: Ammonium
NHS: Nitrogen-hydroxysuccinimide
Ni: Nickel
N-MWCNTs: Nitrogen-saturated multi-walled carbon nanotubes
NPs: Nanoparticles
PAb: Polyclonal antibodies
PAMAM: Phthalocyanine-polyamidoamine
PBS: Phosphate-buffered saline
PECVD: Plasma-enhanced chemical vapor deposition
PNA: Peptide nucleic acid
PDT: Photodynamic treatment
PVA: Polyvinyl alcohol
QD: Quantum dots
rGO: Reduced graphene oxide
RNA: Ribonucleic acid
ROS: Reactive oxygen species
RT-PCR: Reverse transcriptase polymerase chain reaction
RT-qPCR: Reverse transcriptase quantitative polymerase chain reaction
S/N: Signal/Noise
SAb: S antibody
SARS-CoV-2: Severe acute respiratory syndrome coronavirus 2
sc-SWCNTs: Semiconducting single-walled carbon nanotubes
SiO_2_: Silicon dioxide
SIV: Simian immunodeficiency viruses
SWCNT: Single-walled carbon nanotube
TCID_50_: 50% tissue culture infectious dose
TiO_2_: Titanium dioxide
USD: United States Dollar
WHO: World Health Organization

## Conflicts of interest

The authors declare no competing interests.

## Supplementary Material
