# Correction: Recent trends in carbon nanotube (CNT)-based biosensors for the fast and sensitive detection of human viruses: a critical review

**DOI:** 10.1039/d3na90097e

**Published:** 2023-10-06

**Authors:** Hicham Meskher, Hussain Chaudhery Mustansar, Amrit Kumar Thakur, Ravishankar Sathyamurthy, Iseult Lynch, Punit Singh, Tan Kim Han, Rahman Saidur

**Affiliations:** a Department of Process Engineering, Kasdi-Merbah University Ouargla 30000 Algeria hicham.meskher@g.enp.edu.dz; b Department of Chemistry and Environmental Science, New Jersey Institute of Technology Newark 07102 NJ USA; c Department of Mechanical Engineering, KPR Institute of Engineering and Technology Arasur Coimbatore 641407 Tamil Nadu India amritt1@gmail.com; d School of Geography, Earth and Environmental Sciences, University of Birmingham Edgbaston Birmingham B15 2TT UK I.Lynch@bham.ac.uk; e Institute of Engineering and Technology, Department of Mechanical Engineering, GLA University Mathura Uttar Pradesh 281406 India; f Research Centre for Nano-Materials and Energy Technology (RCNMET), School of Engineering and Technology, Sunway University No. 5, Jalan Universiti, Bandar Sunway Petaling Jaya 47500 Malaysia saidur@sunway.edu.my; g Mechanical Engineering Department, King Fahd University of Petroleum and Minerals Dhahran 31261 Saudi Arabia; h Interdisciplinary Research Center for Renewable Energy and Power Systems (IRC-REPS), King Fahd University of Petroleum and Minerals Dhahran 31261 Saudi Arabia

## Abstract

Correction for ‘Recent trends in carbon nanotube (CNT)-based biosensors for the fast and sensitive detection of human viruses: a critical review’ by Hicham Meskher *et al.*, *Nanoscale Adv.*, 2023, **5**, 992–1010, DOI: https://doi.org/10.1039/D2NA00236A.

The authors regret that in the caption of Fig. 1, ref. 36 was wrongly attributed as the original source of the figure. The correct figure caption is shown here:

**Fig. 1** The assembly of a sandwich-type carbon nanotube (CNT) immunosensor and its detection method is depicted schematically. The antibodies are attached onto CNTs through a poly(allylamine) layer. This figure has been adapted/reproduced from ref. 117 with permission from Wiley, copyright 2014.

The Royal Society of Chemistry apologises for these errors and any consequent inconvenience to authors and readers.

## Supplementary Material

